# Characterizing the Roles of *Cryphonectria parasitica* RNA-Dependent RNA Polymerase-Like Genes in Antiviral Defense, Viral Recombination and Transposon Transcript Accumulation

**DOI:** 10.1371/journal.pone.0108653

**Published:** 2014-09-30

**Authors:** Dong-Xiu Zhang, Martin J. Spiering, Donald L. Nuss

**Affiliations:** Institute for Bioscience and Biotechnology Research and Department of Cell Biology and Molecular Genetics, University of Maryland, Rockville, Maryland, United States of America; University of Wisconsin - Madison, United States of America

## Abstract

An inducible RNA-silencing pathway, involving a single Dicer protein, DCL2, and a single Argonaute protein, AGL2, was recently shown to serve as an effective antiviral defense response in the chestnut blight fungus *Cryphonectria parasitica*. Eukaryotic RNA-dependent RNA polymerases (RdRPs) are frequently involved in transcriptional and posttranscriptional gene silencing and antiviral defense. We report here the identification and characterization of four RdRP genes (*rdr1–4*) in the *C. parasitica* genome. Sequence relationships with other eukaryotic RdRPs indicated that RDR1 and RDR2 were closely related to QDE-1, an RdRP involved in RNA silencing (“quelling”) in *Neurospora crassa*, whereas RDR3 was more closely related to the meiotic silencing gene SAD-1 in *N. crassa*. The RdRP domain of RDR4, related to *N. crassa* RRP-3 of unknown function, was truncated and showed evidence of alternative splicing. Similar to reports for *dcl2* and *agl2*, the expression levels for *rdr3* and *rdr4* increased after hypovirus CHV-1/EP713 infection, while expression levels of *rdr1* and *rdr2* were unchanged. The virus-responsive induction patterns for *rdr3* and *rdr4* were altered in the Δ*dcl2* and Δ*agl2* strains, suggesting some level of interaction between *rdr3* and *rdr4* and the *dcl2*/*agl2* silencing pathway. Single *rdr* gene knockouts Δ*rdr1–4*, double knockouts Δ*rdr1/2*, Δ*rdr2/3*, Δ*rdr1/3*, and a triple knockout, Δ*rdr1/2/3*, were generated and evaluated for effects on fungal phenotype, the antiviral defense response, viral RNA recombination activity and transposon expression. None of the single or multiple *rdr* knockout strains displayed any phenotypic differences from the parental strains with or without viral infection or any significant changes in viral RNA accumulation or recombination activity or transposon RNA accumulation, indicating no detectable contribution by the *C. parasitica rdr* genes to these processes.

## Introduction

Mycoviruses and other selfish genetic elements, e.g., transposons, are widely distributed throughout the filamentous fungi. A variety of mechanisms have been identified by which fungi restrict the accumulation and transmission of such elements [Reviewed in 1,2]. The genetic non-self recognition system, vegetative incompatibility, restricts transmission of mycoviruses [3]–[6] and potentially transposable elements [7], [8] between genetically dissimilar individuals in a population. Silencing mechanisms act to control the transcription of transposable elements and accumulation of viruses. Demonstrated fungal transposon silencing mechanisms include repeat-induced point mutations (RIP) [9] and methylation [10]. RNA interference has been shown to restrict both transposon [11]–[15] and virus accumulation in filamentous fungi [16], [17].

The RNA-silencing antiviral pathway is highly conserved among plants, invertebrates and fungi [18], consisting of two core components belonging to the Dicer-like and Argonaute nuclease families [19]. Dicer-like nucleases recognize and, with an associated RNase III-type activity, process viral double-stranded or highly structured RNA into small RNAs of 21–24 nts in length. With the aid of an Argonaute protein, these virus-derived small RNAs (vsRNAs) are subsequently incorporated into an effector complex called the RNA-induced silencing complex (RISC). One strand of the vsRNA is degraded and the remaining strand then serves to guide the effector complex to the cognate viral RNA that is then cleaved by an RNase H-like activity associated with the Argonaute protein. The role of RNA silencing as an antiviral defense mechanism in fungi was first demonstrated for the chestnut blight fungus *Cryphonectria parasitica* [16]. Although *C. parasitica* encodes two Dicer-like genes and four Argonaute-like genes, only Dicer 2 (*dcl2*) and Argonaute 2 (*agl2*) are required for antiviral defense [16], [20]. DCL2 and AGL2 were also shown to contribute significantly to viral RNA recombination [20], [21].

As clearly demonstrated for plants [14], [22], [23] and the nematode *Caenorhabditis elegans* [24]–[26], the Dicer/Argonaute-dependent antiviral RNA-silencing response is further amplified by host RNA-dependent RNA polymerases. Fungal-encoded RdRPs have been reported to play a role in transgene silencing [27]–[28], meiotic silencing [29]–[30], production of qiRNA induced by DNA damage [31], sex-induced silencing [32] and restriction of transposon accumulation [11]–[14]. The role of fungal RdRPs in the antiviral defense response is unknown. The primary goal of this study was to identify and characterize the RdRP genes in *C. parasitica* and examine their contribution to the RNA-silencing antiviral defense response and RNA silencing-mediated promotion of viral RNA recombination.

## Materials and Methods

### 2.1. Fungal strains, growth condition and virulence assay

Wild-type *C. parasitica* strain EP155 (ATCC 38755) was used for generating *rdr1* gene disruption strain Δ*rdr1*. Strain DK80, a mutant of EP155 disrupted in the *ku80* gene for non-homologous end-joining DNA repair to promote integration of homologous DNA sequence [33], was used to generate all other RdRP gene-disruption strains. All fungal cultures were maintained on PDA at 22°C. Viral infection of *C. parasitica* strains was achieved by transfecting viral RNA-coding strand transcripts into fungal spheroplasts by electroporation [34]. The *in vitro* growth of virus-infected and virus-free fungal mycelium of *rdr* gene knockouts and parental strains was assessed on PDA plates; colony diameters were measured after 7 days of growth. Asexual sporulation was measured in cultures grown on PDA for 14 days. Conidia were washed from plates with 1% Tween 20, subjected to serial dilution, counted in a hemocytometer and plated on PDA to test for viability.

Virulence assays were performed on dormant American chestnut tree stems as described by Hillman et al. [35], with six duplicate inoculations per fungal strain. Inoculated stems were kept at room temperature in a glass tank to maintain humidity. Cankers were measured after 21 days.

The sensitivity of wild-type and mutant strains to DNA mutagens was measured with a modified spot test [31]. Conidia were harvested with 0.8% NaCl, filtered through Miracloth, counted in a hemocytometer and diluted with 0.8% NaCl to a concentration of 10^8^/ml. Aliquots of the conidial stock solution were incubated on a shaker for 2 hrs at 60 rpm and 30 C with or without the DNA mutagens histidine (1.5 or 3.5 mg/ml) or hydroxyurea (2 or 4 mg/ml). The treated condial suspensions were taken through serial dilution and 10 µl, each containing 10^6^, 5×10^3^, 5×10^2^ or 50 conidia, was spotted in duplicate on PDA plates containing the concentration of histidine or hydroxyurea used in the original treatment. Colony diameter was measured after 3 days of incubation at room temperature. Average values for the two replica plates were recorded.

### 2.2. Analysis of RdRP genome sequences, gene organization and phylogenetic relationships

The genome sequence assembly of the *C. parasitica* reference strain EP155, annotated with predicted or experimentally verified gene models and protein functions was retrieved from the U.S. Department of Energy’s Joint Genome Institute (JGI; at http://genomeportal.jgi-psf.org/Crypa2/Crypa2.info.html) and interrogated to identify putative RdRP genes. Additional sequence analyses of PCR-amplified cDNAs were performed by Macrogen (Rockville, MD).

Known or putative RdRP protein sequences from all major eukaryotic taxa were retrieved from GenBank or species genome websites. The sequences were entered into NCBI’s Conserved Domain Search tool (at http://www.ncbi.nlm.nih.gov/Structure/cdd/wrpsb.cgi), to identify the protein regions corresponding to the conserved RdRP domain, which were manually extracted and FASTA formatted. Alignments of the RdRP domain sequences were performed with the MUSCLE algorithm (Version 3.8 at http://www.ebi.ac.uk/Tools/msa/muscle/), using the default parameters. The protein alignments were imported into JalView [36] for inspection of alignments and construction of trees with the neighbor-joining method based on percent identity between sequences.

### 2.3. Generation of RdRP gene knockouts

Identified RdRP gene candidates were disrupted by replacing all or large parts of the *rdr* coding sequences (CDS) with selectable marker cassettes *via* homologous recombination, using the DNA transformation protocol developed by Churchill et al [37]. To increase the capacity for producing strains that contain multiple *rdr* gene disruptions, the Cre-*loxP* recombination system [38] was adapted to allow recycling of the limited number of selectable marker genes available for *C. parasitica* transformation [39]. The diagrams in Figs. S1–S3 show the details of the disruption schemes and origins of *rd*r1, *rdr2* and *rdr4* gene disruptions. The disruption scheme for *rdr3* was previously described in Zhang et al. [39]. Overlap-extension PCR was used to generate the gene-disruption fragments comprising the antibiotic resistance gene cassette and flanking sequences of the targeted *rdr* genes. Δ*rdr1* was generated by replacing *rdr1* with a cassette consisting of the *trpC* promoter from *Aspergillus nidulans* in front of the hygromycin B-phosphotransferase (*hph*) gene (Pro_TrpC_-hph) (Fig. S1). Δ*rdr2* was obtained by replacing a 1.5-kb *rdr2* fragment with a 3-kb *hph* cassette (Fig. S2). Δ*rdr3* was generated with a split marker approach using two fragments of a *loxP*-pro_tubB_-*hph*-*loxP* cassette as described in Zhang et al. [39]. Δ*rdr4* was generated by replacing the *rdr4* gene with the intact *loxP*-Pro_tubB_-*hph*-*loxP* cassette (Fig. S3).

**Figure 1:**
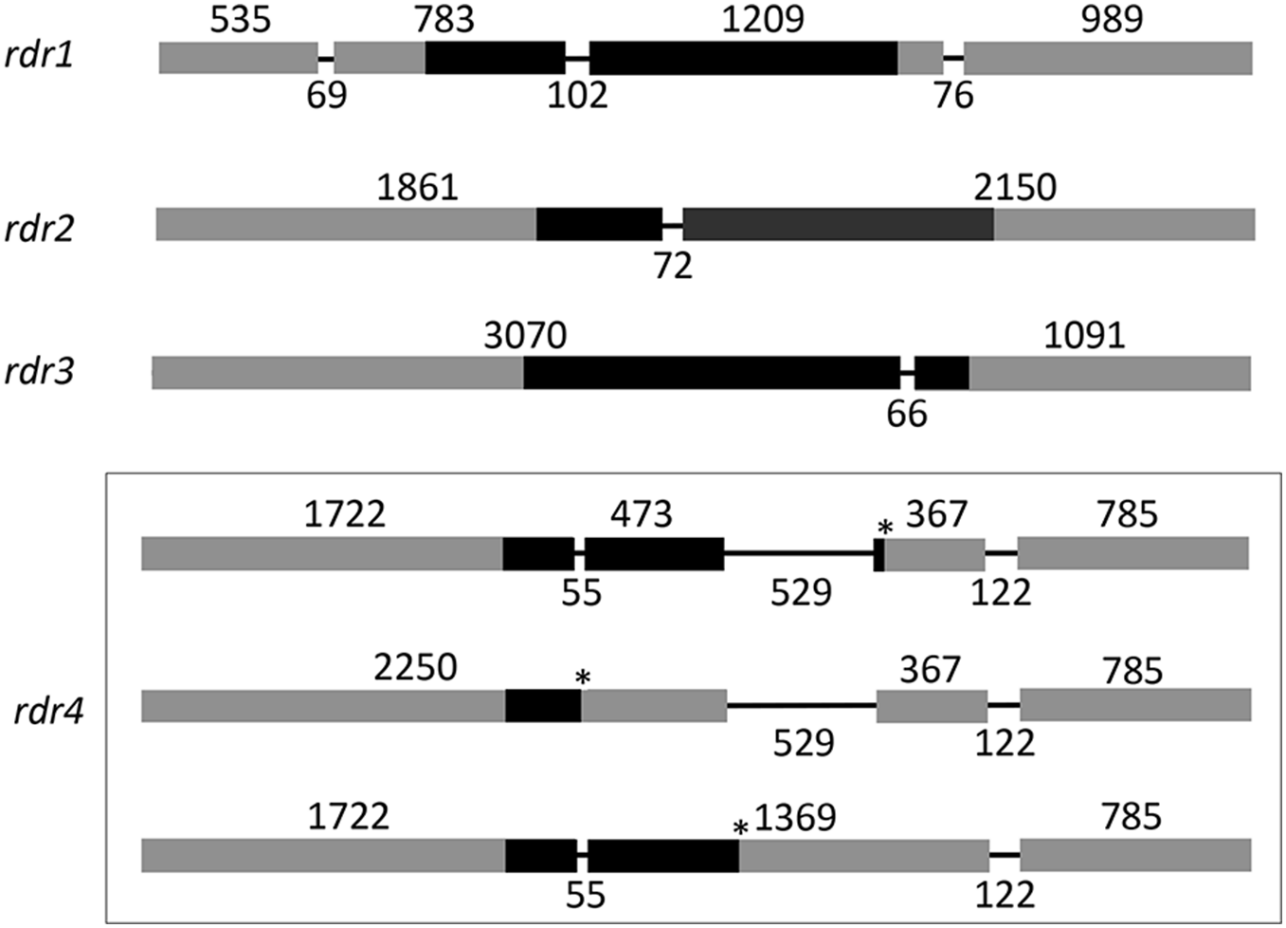
Gene organization of *rdr1*, *rdr2*, *rdr3* and *rdr4*. Positions of exons (boxes) and introns (solid lines) were determined by comparisons of genomic and cDNA sequences for each gene (see text). Black boxes indicate regions corresponding to the RdRP domains in the encoded RDR proteins. Three alternatively spliced sequences were identified in *rdr4*, each predicted to result in a truncated RdRP domain because of premature stop codons indicated by asterisks. Lengths are indicated above and below exons and introns, respectively.

To generate double *rdr* knockout strains, a neomycin (*neo*) gene cassette in pSK666 [20] was cloned into pKAES183 (provided by Dr. Christopher Schardl, University of Kentucky) by restriction-enzyme digestion and T4 enzyme ligation, to replace Pro_tubB_-phleomycin with the *neo* gene behind the *gpd* promoter from *A. nidulans*, resulting in a *loxP*-Pro_gpd_-*neo*-*loxP* construct. The Δ*rdr1/3* and Δ*rdr2/3* double mutant strains were generated by disrupting the *rdr1* or *rdr2* genes with the *loxP*-Pro_gpd_-*neo*-*loxP* cassette in the Δ*rdr3* strain background as described in Zhang et al. [39]. The Δ*rdr1/2* double mutant strain was generated by disrupting the *rdr1* gene with the *loxP*-Pro_gpd_-*neo*-*loxP* cassette in the Δ*rdr2* strain background [described in Fig. 1B of 39]. The Cre-*loxP* system relies on the Cre recombinase to eliminate marker genes flanked by *loxP* sites, thus enabling multiple reuse of the same marker genes for generating serial knockouts [40]. As described in Zhang et al. [39] and included here for convenience, for use in recycling selectable marker genes in *C. parasitica*, a Pro_gpd_–*Cre* gene construct was cloned into pCPX-BSD1 [41], resulting in the construct pCPX-Cre, containing blasticidin as selectable marker. Using the method of Churchill et al. [37], the pCPX-Cre plasmid was transformed into Δ*rdr1/3* and Δ*rdr2/3* spheroplasts for *Cre* expression to eliminate selectable marker genes *via* recombination at the *loxP* sites flanking the *hph* or *neo* marker genes to produce marker gene-free strains Δ*rdr1/3-M* and Δ*rdr2/3-M*. Blasticidin was used at 600 µg/ml for transformant selection on regeneration media; although untransformed colonies also grew at this concentration, they did so at a slower rate than the pCPX-*Cre*-containing transformants. Therefore, putative transformants were selected by their larger colony size to screen for the loss of hygromycin and/or neomycin resistance by replica-plating on three different PDA media: medium containing 50 µg/ml hygromycin PDA, medium containing 25 µg/ml neomycin PDA or antibiotic-free medium. The antibiotic-containing plates were screened for transformed colonies that displayed little or no growth, and the corresponding colonies on the antibiotic-free plates were subjected to single-spore isolation on PDA. Excision of the *neo* and *hph* genes was confirmed by PCR analysis using the same primer pairs used to confirm *rdr* gene knockouts.

Δ*rdr1/2/3* triple knockout strains were engineered in two ways. One strain (F1) was constructed by generating a *rdr1*-disruption fragment containing Pro_TrpC_-*hph* by direct PCR amplification from the disrupted *rdr1* gene in EP155-based disruption strain Δ*rdr1*, the product of which was then used to transform strain Δ*rdr2/3-M*. A second triple knockout strain, C1, was generated in the Δ*rdr1/3-M* strain background by disrupting the *rdr2* gene with a Pro_tubB_-*hph* cassette without flanking *loxP* sites from pKAES173 (provided by Dr. Chris Schardl, University of Kentucky). Transformants were single spored and disruptions of all *rdr* genes were confirmed by PCR.

These extensive disruption studies took advantage of *C. parasitic* strain DK80 derived from reference strain EP155 by disruption of the *ku80* gene for non-homologous end-joining DNA repair to promote homologous DNA recombination [33]. The *rdr1* disruption mutant Δ*rdr1* was generated in strain EP155 prior to the availability of strain DK80. No phenotypic differences have been observed between the DK80 strain and reference strain EP155, whether uninfected or following hypovirus infection. However, strain EP155 was used as the parental control strain for all experiments involving Δ*rdr1* and DK80 was used as the parental control in experiments involving all other individual and multiple *rdr* gene disruption mutants. This resulted in the inclusion of both parental strains in many of the experiments described in this report.

### 2.4. Nucleic acid preparation and analysis

DNA extraction was performed with a small-scale method previously described by Spiering et al. [42]. RNA used for agarose gel analysis of viral full-length and defective interfering (DI) double-stranded RNAs was prepared as described by Sun et al. [20]. Total RNA for reverse-transcription real-time PCR (RT-qPCR) and RNA-seq analysis was extracted with Qiagen’s (Valencia, CA) RNeasy kit. The Monster 1^st^ strand cDNA synthesis kit (Epicentre, Madison, WI) was used for cDNA synthesis from total RNA, following the manufacturer’s protocol, and full-length cDNA copies of each *rdr* gene were amplified by PCR (Takara Tech Enzyme, Takara) and sequenced.

Transcript accumulation for selected genes was measured by one-step reverse-transcription real-time PCR (RT-qPCR), using AgPath-ID RT-PCR reagents (Ambion USA) and an ABI 7300 real-time PCR system. To guard against potential amplification of contaminating genomic DNA in the RNA samples, at least one primer or probe for each *rdr* gene was designed to span an intron/exon boundary, identified by comparison of cDNA and genomic DNA sequences. Primer and probe design and general RT-qPCR methods were performed as described by Zhang et al. [43]. Calculations of transcript accumulation values were performed with the comparative threshold cycle method (ΔΔCt) normalized against the β-tubulin gene transcript levels and calibrated against the normalized value in the wild-type strain. The primer/probe sets for the Dicer-2 and Argonaute-2 genes, *dcl2* and *agl2*, respectively, were the same as described by Sun et al. [20]. Other primers used in this study are shown in Table 1.

**Table 1:** Primers and probes used in this study.

Primer name	Sequence (5′→ 3′)	Note
R1f	AAC CCG CAC GAG ACA CAG TGG GCA GAT	Δ*rdr1* (*Pro_trpC_-hph*) check
R1r	GTG TGG AGC AGC AGA TGG AGA AAT ATG	
R2f	ATG AAC ACT TCC CAG GGC CA	Δ*rdr2* (hph cassette) check
R2r	TCA ATC GTC CTC GTC CAA CTC	
R4f	GTC CTT GTA GAC CAT GTG TGT CCC TT	Δ*rdr4* (*loxP-Pro_tubB_-hph-loxP*) check
R4r	TCG GAC CCC AGG GTA ACC ACC GCC AAC GTG	
TQMrdr1F	AGA TCT CCA AGT TGA GCA CGG GAA	*rdr1* gene expression level assay with RT-qPCR
TQMrdr1R	TCG CAG ACC CGA TAG CAG TGA TAA	
TQMrdr1probe	/56-FAM/AAA GCT CCT/ZEN/CGG ATC CTG TAC ATC GT/3IABkFQ/	
TQMrdr2F	GGT CCC AGC CTG CAT TAA AGT TGT	*rdr2* gene expression level assay with RT-qPCR
TQMrdr2R	TCA TCT GGC TGG GTT CGA AGA TGA	
TQMrdr2probe	/56-FAM/TTG TCC TCA/ZEN/GCA GAA CTA GCC CGA CA/3IABkFQ/	
TQMrdr3F	AAA CGA CTC TCT GGG ACT CAT AGC CA	*rdr3* gene expression level assay with RT-qPCR
TQMrdr3R	GGA ATT TGG CAA GCT CAA GGC ACT	
TQMrdr3probe	/56-FAM/ACA AGG CCT/ZEN/GGG CGG ACA AA/3IABkFQ/	
TQMrdr4F	TGG CGA GGA GCT TGA CCA ACA TAA	*rdr4* gene expression level assay with RT-qPCR
TQMrdr4R	CAG CGA CTG AAG CGT TGG TTG AAA	
TQMrdr4probe	/56-FAM/ACG AAA CTC/ZEN/CAA CAC CGC CAG ATC T/3IABkFQ/	
rdr1d1	ATG CCG CCG CGA AAA TCA TT	full-length cDNA amplification
rdr1u1	TCA ATC ATC CTC TTG CTG GCT	
rdr2d1	ATG AAC ACT TCC CAG GGC CA	
rdr2u1	TCA ATC GTC CTC GTC CAA CTC	
rdr3d1	ATG ACC ACC CCT ACC AGG GCC CCT AA	
rdr3u1	TTA GTG CTC ATC ACG TAC GCC AGT A	
rdr4d1	ATG GAG GTC CAC ATG CAC	
rdr4u1	TCA TTT ACC ACT GCA GCC	

### 2.5. Viral recombination assay

All *rdr*-knockout, DK80 and Δ*dcl2* strains were transfected with a replication-competent, GFP-containing viral vector Ctp40[2AEGFP] [44] by transfection with spheroplasts [34]. One week after the transfection, each infected strain was transferred to PDA and then sub-cultured to new PDA plates every 5 days for up to 6 transfers. Total RNA was isolated from sub-cultured colonies to monitor for presence of defective interfering viral dsRNAs and to serve as template to synthesize cDNA for PCR analysis with primer pair CHV1-2KF/CHV1-3KR [45] to monitor for maintenance of the EGFP coding domain in the viral vector RNA. GFP accumulation in infected fungal hyphae was also monitored under a fluorescence stereomicroscope (Model MVX10; Olympus) [21].

### 2.6. RNA-seq analysis of transposon expression

To identify and extract transposon sequences from the *C. parasitica* genome assembly at JGI (http://genomeportal.jgi-psf.org/Crypa2/Crypa2.home.html), the keywords of all gene models were queried with the following search terms: “transposon,” “retrotransposon,” “transposase,” “transposable,” “transposable element,” “copia,” “FOT,” “Flipper,” “gag,” “gag-pol,” “gypsy,” “LINE,” “pogo” and “SINE.” This search identified >500 gene models whose IDs and associated annotations were downloaded as csv files and sequences as FASTA files. Since these searches frequently resulted in repeated retrieval of the same sequences, the retrieved sequences IDs were manually checked and all duplicate sequences removed. Because many transposon copies in fungal genomes are highly similar in sequence–potentially affecting the read mappings in the RNA-seq analyses–all of the retrieved sequences were aligned with each other in BLASTN. Those sequences having >99% identity along >95% of their total length in the BLAST alignment were then combined as a single sequence consensus in JalView [36] for further analysis. In addition, sequences <500 bp long were excluded from the analysis, resulting in a final set of 104 sequences. Of these, 71 were identified by their annotations in JGI or by BLASTX searches as class I transposable elements (i.e., retroelements or retrotransposons), 21 as class II (DNA) transposons and 12 sequences were similar to RNA-associated or hypothetical proteins (Table S1).

To quantify expression from each of the transposon sequences, total RNA was extracted from the *rdr* gene knockouts Δ*rdr1/2/3(C1)*, Δ*rdr1/2* and Δ*rdr4* and the DK80 parental strain from mycelium grown on PDA for 7 days. Three biological replicates were used for Δ*rdr1/2/3* and Δ*rdr1/2* and analyzed separately by RNA-seq. One biological sample was used for Δ*rdr4*. The RNA was submitted to the Institute for Bioscience and Biotechnology Research DNA Sequencing Facility for construction of sequencing libraries containing cDNA fragments with an average length of 200 nucleotides. Paired-end reads sequencing data were acquired on an Illumina HiSeq 1000 instrument. On average, >40 million sequence reads were obtained per sample. The reads were uploaded into CLC Genomics Workbench 6.0 (CLCBio, Aarhus, Denmark) and mapped to the transposon sequences for RNA-seq analysis. The following (default) settings were used in the read mappings: a minimum length fraction of 0.9, minimum similarity fraction of 0.8 and maximum number of hits per read of 10; the minimum and maximum distances between paired reads were set at 100 and 250, respectively. Expression was estimated as reads per kb of coding sequence per million mapped reads (RPKM). The RNA-seq expression data from *rdr* gene knockouts and the DK80 control were used to calculate fold changes in expression by ratio-based analysis to identify differentially expressed transcripts from transposon sequences in each *rdr* gene knockout strain. Raw RNA-seq data have been deposited in the NCBI sequence read archive (SRA) under BioProject ID PRJNA258639.

## Results

### 3.1. Identification and features of RdRP-like genes in C. parasitica strain EP155

The *C. parasitica* genome sequence assembly generated by the U.S. Department of Energy’s Joint Genome Institute for reference strain, EP155, was used as a resource for RdRP gene identification in this study. To identify RdRP gene candidates, the keywords in the annotated EP155 genome sequence were queried with “RdRP” as search term. This search yielded four RdRP-like sequences. In addition, a BLASTP search of the EP155 sequence was conducted, using QDE-1, the RdRP required for transgene silencing (Quelling) in *Neurospora crassa* [27], [46], as query, which did not result in any additional sequences. The RdRP-like genes identified were designated *rdr1* (scaffold_11∶693898–697090 in JGI), *rdr2* (scaffold_1∶365214–369296), *rdr3* (scaffold_5∶3858759–3862568) and *rdr4* (scaffold_4∶4500867–4504921). The genomic sequences from each putative RdRP gene were then used to obtain cDNA amplicons by RT-PCR and sequencing. The PCR-amplified cDNAs for *rdr1*, *rdr2* and *rdr3* gave a single product as judged by agarose gel analysis, and intron and exon positions in each of the three genes were identified by sequence comparison with the cognate genomic DNA sequences (Fig. 1). *rdr1* had three introns and four exons, whereas *rdr2* and *rdr3* each contained only one intron and two exons. The location within the coding domain of the second intron in *rdr1* corresponded to that of the only intron in *rdr2* as judged by alignment of the encoded amino acid sequences relative to the exon–intron boundaries in the two genes. No such correspondence was seen for the only intron in *rdr3*.

Amplification of the full-length ORF in *rdr4* gave multiple cDNA bands. To determine the reason for this potential diversity in *rdr4* transcripts, the most prominent three bands were individually gel purified and sequenced. This analysis revealed that the variable banding pattern had most likely originated from alternative splicing events in the *rdr4* gene. *rdr4* contained three introns, and the first two introns were either spliced out or remained unspliced (Fig. 1). The four *rdr* genes have the following GenBank accession numbers: *rdr1* (HF912382); *rdr2* (HF912383); *rdr3* (HF912384); *rdr4* (HF912385).

**Figure 2:**
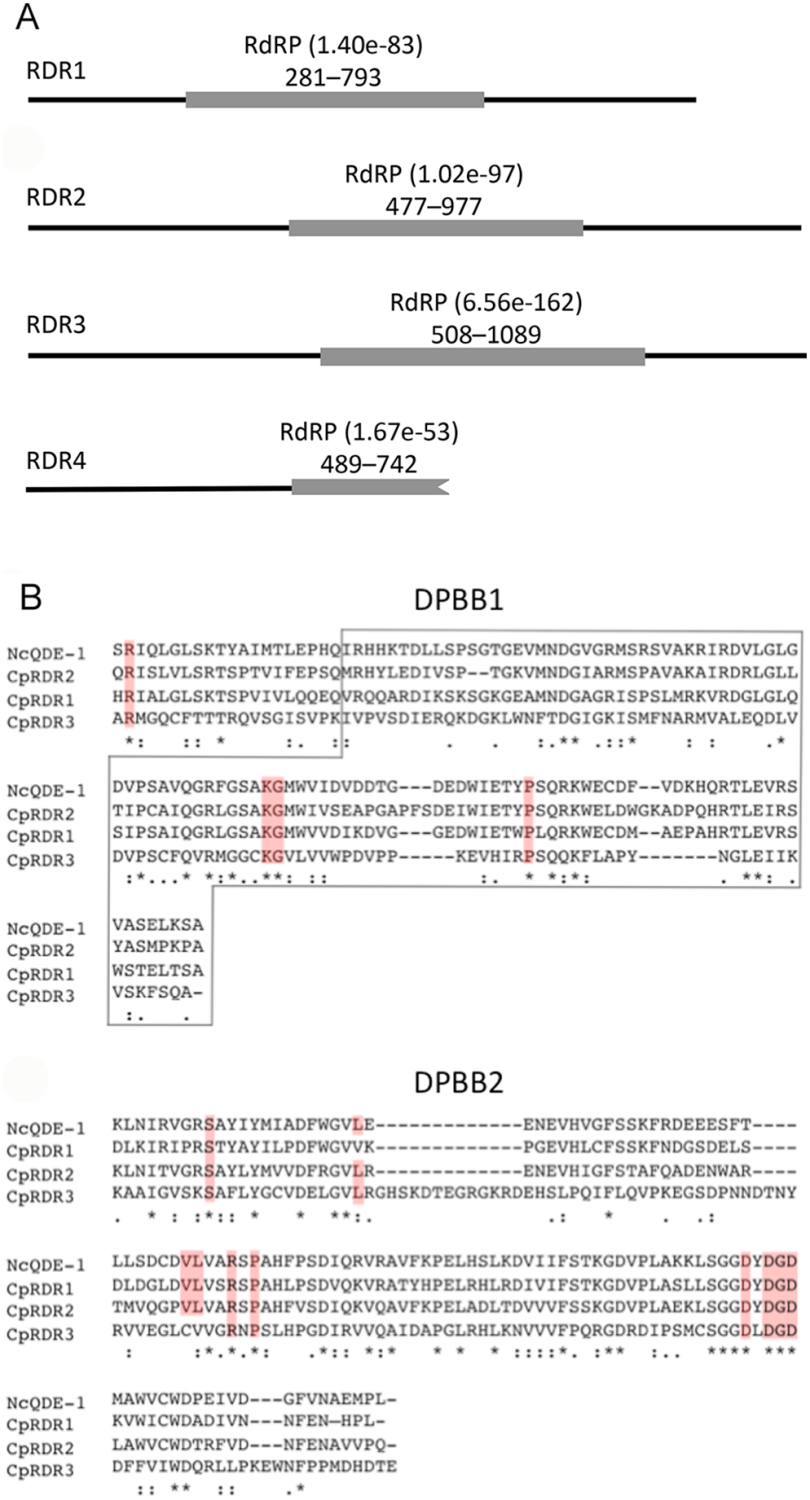
Conserved RdRP domains and amino acid sequences of *C. parasitica rdr* genes. **A**). The RdRP domains were identified by searching NCBI’s Conserved Domains database (http://www.ncbi.nlm.nih.gov/Structure/cdd/wrpsb.cgi) and are indicated by grey boxes; solid lines represent full-length proteins. E-values of each RdRP domain match and the range of amino acids included in the match are indicated next to and below the domain name, respectively. The longest predicted RDR4 protein is indicated here and contains an RdRP domain that is truncated due to a premature stop codon in its gene transcript and that lacks a conserved DPBB domain. **B**). The three *C. parasitica* RDRs containing complete RdRP domains were included in an alignment with QDE-1 from *N. crassa*, an RdRP involved in RNA silencing (Quelling); highly conserved DPBB domains and residues in QDE-1 [47] are indicated by boxes and are highlighted in red, respectively.

Searches for conserved protein domains with the CD-Search Tool at NCBI (at http://www.ncbi.nlm.nih.gov/Structure/bwrpsb/bwrpsb.cgi) revealed that RDR1, RDR2 and RDR3 each contained an RdRP-type conserved domain (pfam ID: pfam05183; E-values <10^−80^). Interestingly, the *rdr4* gene did not contain an intact RdRP domain; the predicted RDR4 protein sequence of the spliced transcripts contained in all cases a disrupted RdRP domain (Fig. 2A) that lacked the DXDGD signature motif of eukaryotic RDRs (Fig. 2B).

QDE-1 from *N. crassa* is involved in RNA silencing (Quelling) [27] and several key amino acid residues and domains required for its activity have recently been identified [47]. To see if the *C. parasitica* RdRPs also contained these residues and domains, we conducted comparative sequence analysis of RDR1– RDR3 with QDE-1. Since *rdr4* did not appear to encode a functional RDR protein, it was not included in the analysis. All three *C. parasitica* RDRs contained three invariant aspartic acid residues, corresponding to D1007, D1009 and D1011 in QDE-1 in its catalytic double-psi-β-barrel (DPBB2) domain [47] that constitute the DXDGD motif essential for catalyzing the formation of phosphodiester bonds during RNA synthesis [47]. Furthermore, all three RDRs contained three absolutely conserved residues present in the DPBB1 domain of QDE-1 (Fig. 2B). Including the DXDGD motif, ten highly conserved amino acid residues are present in the DPBB2 domain of QDE-1 [48], all of which were also present in RDR2; RDR1 shared nine and RDR3 eight out of the ten highly conserved residues in QDE-1. This analysis indicated that RDR1– RDR3 possess the active-site residues required for RDR activity; it also revealed that the RDR1 and RDR3 sequences were more divergent from QDE-1 than RDR2 in highly conserved RDR regions.

BLAST sequence alignments (all having E-values <10^−19^) of the full-length RdRP sequences supported the above pattern of divergence among the three RdRPs: whereas QDE-1 aligned with RDR2 at 42% identity (59% similarity) along 1,125 amino acids, its alignment with RDR1 gave 40% identity (56% similarity) along 1,014 residues, indicating a somewhat greater level of divergence between RDR1 and QDE-1 (alignments not shown). RDR3 failed to give a contiguous alignment with QDE-1, and resulted in only 26% identity (43% similarity) along 340 residues of the longest aligned region. These analyses suggested that out of the three likely functional RdRP-like proteins in *C. parasitica*, RDR2 appeared to be the one most closely related to QDE-1. However, despite this relatively close relationship, RDR2 displayed only moderate (<60%) similarity to QDE-1, suggesting the possibility of some functional divergence between the two proteins. None of the three *C. parasitica* RDRs contained an RNA-recognition motif (RRM; pfam ID: cd00590); such RRM domain was identifiable in *N. crassa* SAD-1 (meiotic silencing), but not in QDE-1, suggesting that the RRM motif is not essential for RNA silencing activity.

### 3.2. Sequence relationships of RdRP-like proteins from C. parasitica with other RdRP protein sequences

**Figure 3:**
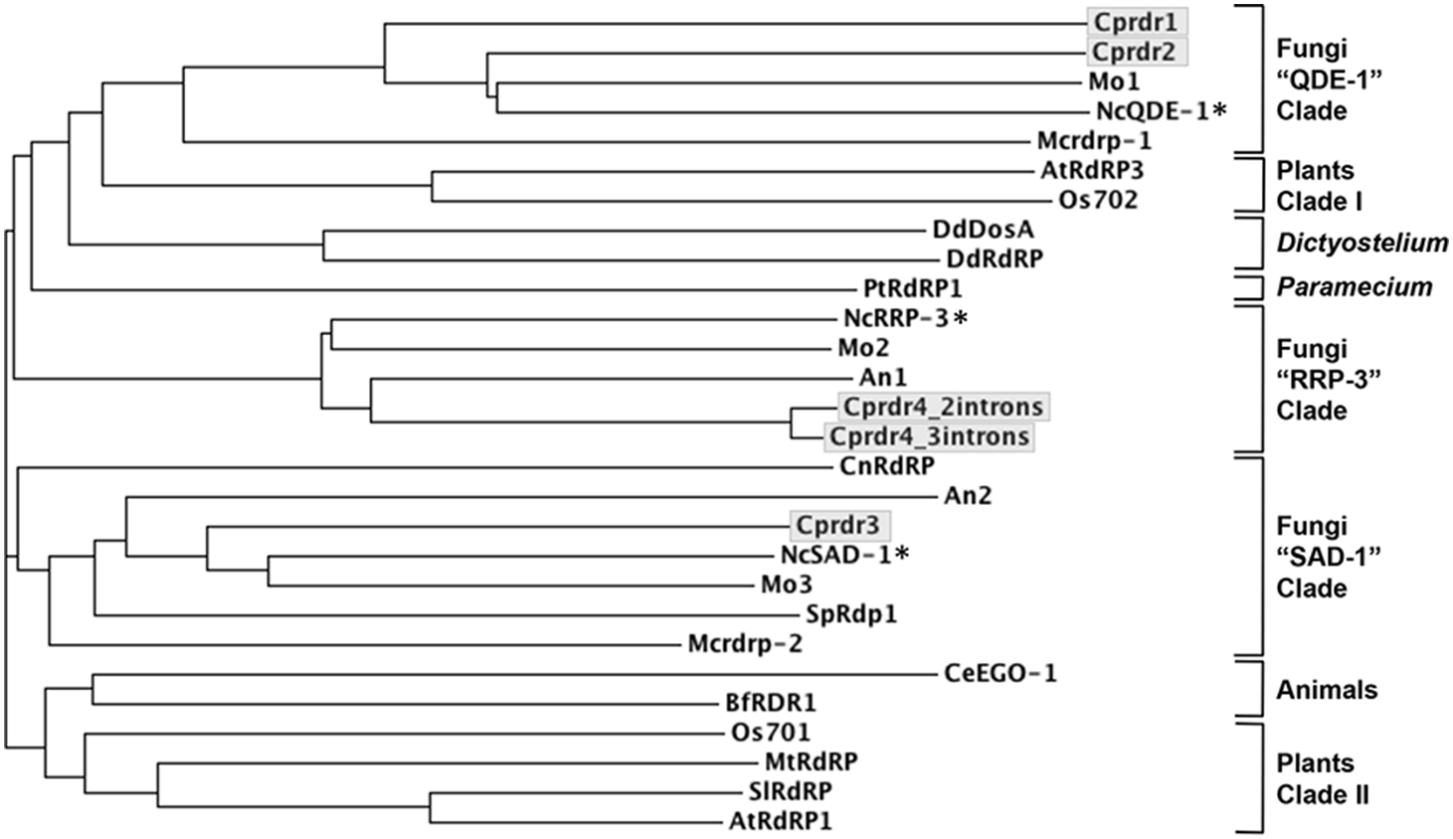
Sequence relationships of *C. parasitica* RDR-like proteins with putative and known RDRs in other eukaryotes. RdRP domains were extracted from each sequence and aligned for tree reconstruction as described in the Methods. The four RDR-like *C. parasitica* sequences are shown in shaded boxes; two alternatively spliced forms of RDR4, identified by cDNA sequencing (resulting in a truncated RdRP domain) are shown. The RDRs in *N. crassa* are indicated by asterisks and were used to name the three fungal RDR clades, indicated on the right, along with the clades containing RDR sequences in other eukaryotes. Sequence accessions and species names are the following: An1 = AN2717 (*Aspergillus nidulans*); An2 = AN4790 (*A. nidulans*); AtRdRP1 = NP_172932.1, RNA-dependent RNA polymerase 1 (*Arabidopsis thaliana*); AtRdRP3 = NP_179581.2, RNA-dependent RNA polymerase-like protein (*A. thaliana*); BfRDR1 = AAQ10792.1, RNA-directed RNA polymerase-like protein (*Branchiostoma floridae*); CeEGO-1 = NP_492132.1, Protein EGO-1 (*Caenorhabditis elegans*); CnRdRP = ACJ04634.1, RNA-dependent RNA polymerase (*Cryptococcus neoformans var. grubii*); Cprdr1 = HF912382, putative RNA-dependent RNA polymerase (*Cryphonectria parasitica*); Cprdr2 = HF912383, putative RNA-dependent RNA polymerase (*C. parasitica*); Cprdr3 = HF912384, putative RNA-dependent RNA polymerase (*C. parasitica*); Cprdr4 = HF912385, putative rdr4 pseudogene (*C. parasitica*); DdDosA = AF117611_1, DosA protein (*Dictyostelium discoideum*); DdRdRP = EAL62541.1, RNA-directed RNA polymerase (*D. discoideum*); Mcrdrp-1 = JGI scaffold_05∶2640291–2644233 (*Mucor circinelloides*); Mcrdrp-2 = JGI scaffold_05∶245898–249911 (*M. circinelloides*); Mo1 = EHA51817.1, RNA-dependent RNA polymerase (*Magnaporthe oryzae*); Mo2 = EHA52286.1, RNA-dependent RNA polymerase (*M. oryzae*); Mo3 = EHA46264.1, RNA-dependent RNA polymerase 1 (*M. oryzae*); MtRdRP = XP_003608944.1, RNA-dependent RNA polymerase (*Medicago truncatula*); NcQDE-1 = AJ133528, qde-1 gene (*Neurospora crassa*); NcRRP-3 = XP_963405, hypothetical protein (*N. crassa*); NcSAD1 = AAK31733, suppressor of ascus dominance (*N. crassa*); Os701 = NP_918046.1, putative RNA-directed RNA polymerase (*Oryza sativa*); Os702 = NP_913147.1 (*O. sativa*); PtRDRP1 = CAI39057.1, RNA-dependent RNA polymerase (*Paramecium tetraurelia*); SpRdp1 = CAB11093.1, RNA-directed RNA polymerase Rdp1 (*Schizosaccharomyces pombe*); SlRdRP = CAA71421.1, RNA-directed RNA polymerase (*Solanum lycopersicum*).

To gain some broader insight into the relationship of the *C. parasitica* RdRP-like sequences with putative and confirmed RdRP sequences from other species, a multiple sequence alignment of the RdRP-like *C. parasitica* sequences with those from a range of taxa from plants, protists, animals and other fungi was performed. The alignment was restricted to the predicted RdRP domains in each protein, and used to reconstruct a tree. This yielded several major clades, each represented by RDR sequences originating from a single kingdom (Fig. 3). Consistent with the general topology of RdRP trees in previous reports [49], RdRPs from animals grouped in a single well-defined clade, whereas those from fungi, plants and protists assembled in multiple clades, homogeneous for each organismal group. Interestingly, each of the three fungal RdRP clades contained an RdRP from *N. crassa*, i.e., QDE-1 (Quelling), SAD-1 (meiotic silencing) or RRP-3 (function unknown), a pattern that was closely tracked by the four RdRP-like sequences from *C. parasitica*. RDR2 was very closely affiliated with QDE-1, whereas the position of RDR1, although residing in the same (“QDE1”) clade suggested a more distant relationship with both RDR2 and QDE-1. RDR4 (included as two different predicted sequences inferred from alternative splicing and theoretical prediction) was affiliated with RDRs clustering with RRP-3, while RDR3 resided in another fungal clade that included SAD-1. These sequence relationships among the fungal RdRPs were reproducibly reconstructed with additional tree-building methods and evolutionary models (not shown). This analysis suggested distinct biological roles for the three functional *C. parasitica* RdRPs. For example, the affiliation with QDE-1 suggested involvement of RDR2 and possibly RDR1 in posttranscriptional silencing, whereas clustering of RDR3 with SAD-1, an RDR involved in meiotic silencing [29], might suggest that RDR3 functions in a similar, but as-yet unreported, pathway in *C. parasitica*. A possible function for RDR4 was less clear, since it lacked a complete RdRP domain and no functional roles have yet been ascribed to its closest relative in *N. crassa*, RRP3. The analysis thus supported an early split of the four putative *C. parasitica* RdRP-like sequences, very likely preceding the speciation events that had resulted in the species shown in the tree.

### 3.3. Expression of rdr3 and rdr4 is induced upon infection with CHV1-EP713 and suppressed by the viral p29 protein

**Figure 4:**
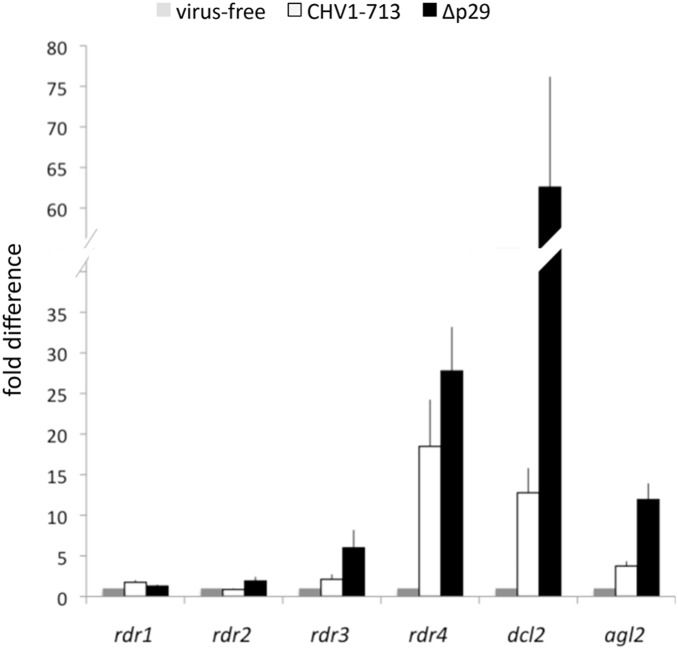
Accumulation of *C. parasitica rdr* transcripts in response to infection by hypovirus CHV-1/EP713 and the CHV-1/EP713 mutant Δp29 that lacks the p29 suppressor of RNA silencing. The relative levels of *rdr1–4* gene transcripts were measured by quantitative real-time RT-PCR (2^−ΔΔ_CT_^ method) for uninfected (grey bars), CHV-1/EP713-infected (open bars) and Δp29-infected (black bars) *C. parasitica* strain EP155. The induction patterns for *dcl2* and *agl2* transcript accumulation has been previously reported [20], [21] and were also measured in parallel here. The identities of the transcript being measured are indicated at the bottom of the histogram. The fold differences in transcript accumulation are indicated on the Y axis and were calculated using the comparative threshold cycle (ΔΔCT) normalized against *tub*β transcript levels and calibrated against the normalized value in the wild-type strain, with standard deviations, based on three independent measurements of two independent RNA preparations, indicated by the error bars. Transcript levels for uninfected *C. parasitica* were set to a value of one.

Expression of *dcl2* and *agl2* involved in the *C. parasitica* RNA silencing antiviral defense response is induced following hypovirus infection and suppressed by the virus-encoded RNA-silencing suppressor, papain-like protease p29 [20]. Thus, if one or more of the *rdr* genes were involved in anti-viral defenses, *rdr* expression would also be expected to be induced in response to viral infection and suppressed by p29. Therefore, the influence of infections by hypovirus CHV-1/EP713 and CHV-1/EP713 in which p29 was deleted (Δp29) on *rdr* gene expression was examined. Expression of *rdr3* and *rdr4* was significantly increased upon viral infection, and this response was greater when strain EP155 was infected by Δp29 (Fig. 4). By contrast, *rdr1* and *rdr2* expression showed only very minor responses to infection with CHV-1/EP713 or Δp29. Expression of *rdr4* showed the strongest upregulation, similar to or even exceeding upregulation of *dcl2* and *agl2* in response to CHV1-EP713. These results indicated that expression of *rdr3* and *rdr4* was significantly induced by hypovirus infection, a response that was attenuated by the viral p29 RNA-silencing suppressor, thus exhibiting transcriptional responses that were somewhat similar to those of the anti-viral defense genes, *dcl2* and *agl2*.

### 3.4. Virus-induced expression patterns for rdr3 and rdr4 are altered in Δagl2 and Δdcl2 strains

**Figure 5:**
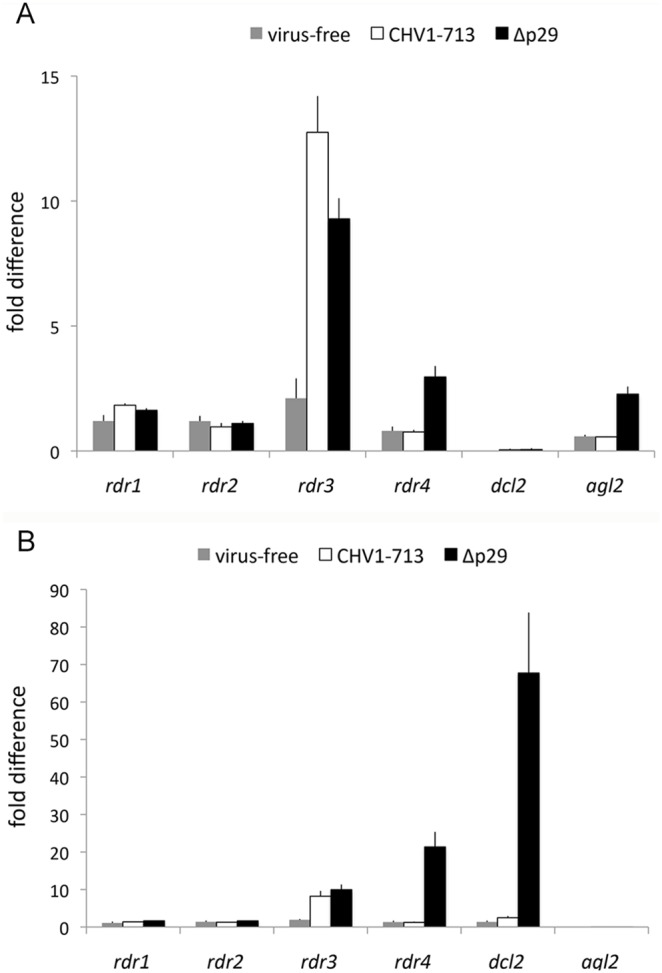
Accumulation of *rdr* transcripts in response to virus infection in *dcl2* and *agl2* disruption mutant strains. **Panel A.** Transcript levels for *rdr1–4* and *agl2* genes were measured in uninfected (grey bars), CHV-1/EP713- (open bars) and Δp29- (black bars) infected Δ*dcl2* disruption strain as described for Figure 4. **Panel B.** Transcript levels for *rdr1–4* and *dcl2* genes were measured in uninfected (grey bars), CHV-1/EP713- (open bars) and Δp29- (black bars) infected Δ*agl2* disruption strain as described for Figure 4.

To investigate whether the expression of *rdr* genes may be affected by the deletion of the antiviral genes *dcl2* or *agl2,* the strains Δ*dcl2* and Δ*agl2* generated in previous studies [20], [21] were examined for *rdr* gene expression. Each strain was infected with CHV-1/EP713 or Δp29 and gene expression analyzed by RT-qPCR. Expression of *rdr1* and *rdr2* in virus-infected and virus-free strains Δ*dcl2* and Δ*agl2* (Fig. 5A–B) was largely similar to expression in strain EP155 (Fig. 4), suggesting little or no effect of the anti-viral gene deletions on expression of *rdr1* and *rdr2*. In contrast, *rdr3* expression was increased ≥10-fold upon virus infection in both Δ*dcl2* and Δ*agl2*, much greater than the 2 to 6-fold increase in expression of *rdr3* in virus-infected EP155. *rdr4* expression was only marginally (≤2-fold) increased in both Δ*dcl2* and Δ*agl2* in response to CHV1-EP713 infection, down from ∼20-fold upregulation in CHV1-EP713-infected EP155. The level of upregulation of *rdr4* expression in Δp29-infected Δ*agl2* was largely similar to that in Δp29-infected EP155, whereas deletion of *dcl2* markedly decreased *rdr4* upregulation to ∼3fold, compared to the almost 30-fold upregulation of *rdr4* in Δp29-infected EP155. Expression of *rdr1* and *rdr2* did not exhibit any detectable changes. These results suggested interactions between *rdr3* and *rdr4* expression and the induction of the *dcl2*/*agl2* RNA silencing pathway in response to virus infection.

### 3.5. Disruption of RdRP genes and testing of contributions to the RNA silencing antiviral defense response

**Figure 6:**
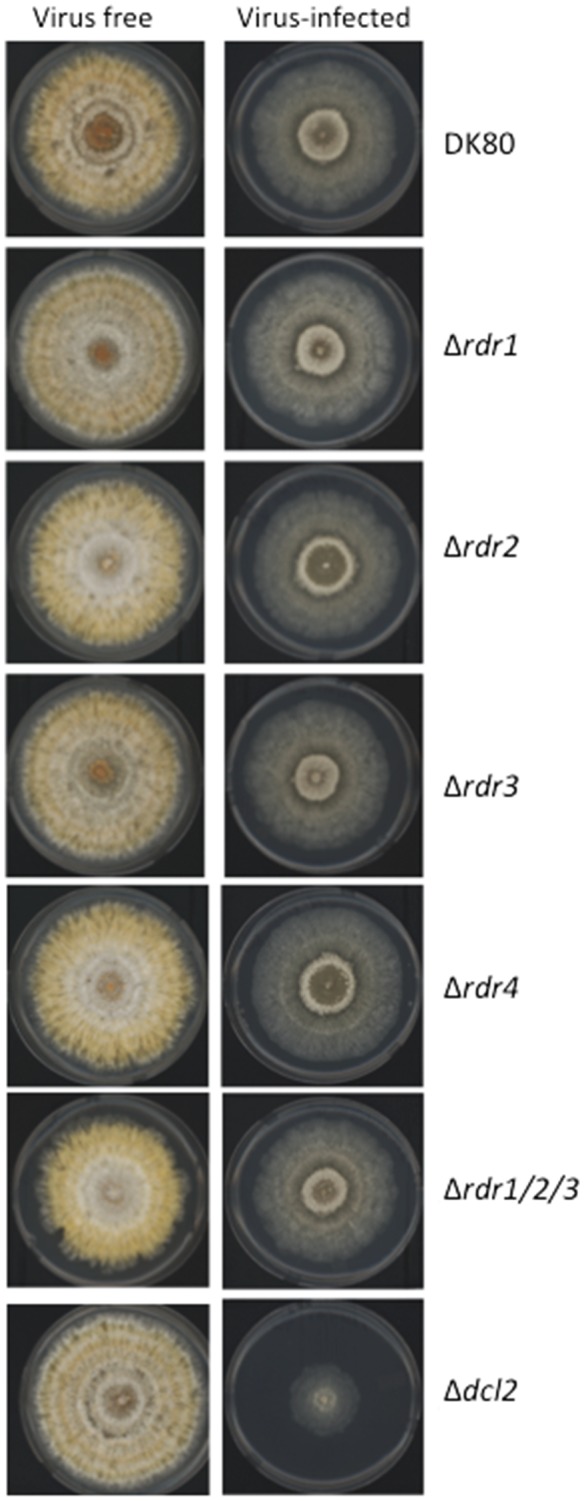
Effect of *rdr* gene disruption and hypovirus CHV-1/EP713 on *C. parasitica* colony growth and morphology. The colonies of uninfected parental strain DK80 and gene disruption mutant strains are shown in the left column while the corresponding hypovirus CHV-1/EP713- infected strains are shown in the right column. The uninfected triple *rdr* mutant strain Δ*rdr1/2/3* and uninfected Δ*dcl2* strains are shown at the bottom of the left column while the corresponding CHV-1/EP713-infected strains are shown in corresponding positions in the right column. Note the severe growth and morphological changes exhibited by the CHV-1/EP713-infected Δ*dcl2* strain. Cultures were grown for 7 days on PDA.

To further investigate the potential roles of the *C. parasitica* RdRP genes in the RNA silencing antiviral defense response, all four *RdRP* genes were disrupted individually and in combination. *C. parasitica* strains carrying disruptions of the antiviral genes *dcl2* and *agl2* became severely debilitated following hypovirus infection [16], [20]. So it was a reasonable expectation that disruption of an RdRP gene involved in the RNA silencing antiviral defense pathway would likewise result in similar readily detectable phenotypic changes. The single Δ*rdr* disruption strains each showed growth phenotypes that were essentially identical to the phenotype of the parental strains both in the absence and presence of infecting hypovirus CHV-1/EP713 (Fig. 6).

In view of these results and to account for the possibility that the RdRP genes have some functional redundancy, a series of double- and triple-disruption mutant strains was generated as described in the methods. *rdr4* was not included in this analysis, since, as shown above, its predicted protein product lacks a functional RdRP domain. Double and triple Δ*rdr1/2/3* knockout strains also exhibited normal growth and colony morphology (examples shown in Fig. 6). Additional analysis revealed no reduction in growth of the multiple *rdr* knockout strains in a virulence assay on inoculated dormant chestnut stems relative to the parental strain or any change in the production of asexual spores (data not shown). *N. crassa qde-1* mutant strains showed sensitivity to DNA damaging agents [31]. In contrast, the *C. parasitica* double and triple *rdr* mutant strains were as sensitive as the wild-type strain to increasing concentrations of histidine and hydroxyurea (Table 2). The combined results for all single, double and triple *rdr* knockout strains revealed no essential role for the *rdr* genes in fungal growth, asexual sporulation, virulence or response to DNA damage-related stress.

**Table 2:** Colony diameter (cm) in DNA-damage sensitivity assay measured after 3 days of growth.

Treatment	PDA only				1.5 mg/ml histidine				3.5 mg/ml histidine				2 mg/ml hydroxyurea				4 mg/ml hydroxyurea			
Spores	10^6^	5×10^3^	5×10^2^	50	10^6^	5×10^3^	5×10^2^	50	10^6^	5×10^3^	5×10^2^	50	10^6^	5×10^3^	5×10^2^	50	10^6^	5×10^3^	5×10^2^	50
EP155	3.5	4.5	4.7	4.2	4.8	4.7	4.6	4.1	3.4	3.8	3.9	3.5	2.3	2.0	1.8	na+	1.3	na	na	na
DK80	3.5	3.9	4.0	3.8	3.4	3.2	3.1	2.8	2.8	3.2	3.1	2.8	2.5	2.0	1.4	na	1.6	na	na	na
Δ*dcl2*	4.9	4.7	4.1	4.3	3.2	4.1	4.2	3.8	3.5	3.7	3.5	3.2	2.3	1.6	1.4	na	2.0	na	na	na
Δ*rdr1/2*	3.8	4.4	4.5	3.9	3.4	4.1	3.7	3.3	3.2	3.4	3.3	2.9	2.4	2.0	1.7	na	1.3	na	na	na
Δ*rdr1/3*	3.2	4.5	3.6	3.8	3.7	3.7	3.5	3.2	3.0	3.0	3.1	3.0	2.6	2.2	1.5	na	1.9	na	na	na
Δ*rdr2/3*	3.9	4.6	3.7	3.7	3.9	3.7	3.4	3.4	3.6	3.5	3.4	3.1	2.2	2.1	1.7	na	1.6	na	na	na
Δ*rdr1/2/3*	3.4	4.1	4.5	4.0	3.5	3.3	3.0	3.2	3.2	3.1	nd*	nd	2.1	2.2	1.5	na	1.5	na	na	na
Δ*rdr4*	3.7	4.6	4.3	4.4	3.7	4.3	4.0	3.6	3.0	3.5	3.3	3.0	2.1	2.2	1.6	na	1.6	na	na	na

*nd, not determined due to contamination on at least one plate.

+ na, not available due to the poor growth of colonies.

CHV-1/EP713 infection of the double and triple *rdr* mutant strains resulted in the standard phenotypic changes that were indistinguishable from those of the CHV-1/EP713- infected parental strains (examples shown in Fig. 6). In contrast, growth of the CHV1-EP713-infected Δ*dcl2* strain used as a control for deletion of antiviral genes in this experiment exhibited a highly debilitated growth phenotype (Fig. 6), as previously described [16]. Moreover, viral RNA accumulation levels were not significantly different between parental and *rdr* mutant strains (data not shown). Based on the combined results, the *C. parasitica rdr* genes appear to play no significant role in the RNA silencing antiviral defense response.

### 3.6. Contribution of host RdRPs to viral recombination

The *C. parasitica* RNA-silencing antiviral defense pathway was shown to contribute to viral RNA recombination [reviewed in 50]. Defective interfering (DI) RNAs that are readily formed in CHV-1/EP713-infected strain EP155, fail to form in either Δ*dcl2* or Δ*agl2* mutant strains [20], [21]. Moreover, non-viral sequences that are rapidly deleted from recombinant hypovirus RNA virus vectors in wild type *C. parasitica* are stably maintained over prolonged periods of sub-culturing in the Δ*dcl2* and Δ*agl2* strains [20], [21]. The collection of *rdr* gene-disruption mutant strains was tested for their ability to support viral RNA recombination. Mutant and control strains were infected with hypovirus CHV-1/EP713 and independently with recombinant hypovirus Ctp40[2AEGFP] that expresses the enhanced green fluorescent protein (EGFP). The infected strains were sub-cultured every 5 days and monitored for fluorescence, maintainence of the EGFP sequence and production of DI RNAs. Fluorescence disappeared in all independent *rdr* gene-disruption mutants and in the Δ*rdr1/2/3* triple mutant within three subcultures, similar to what was observed for the control-infected parental strain. In contrast, fluorescence was retained in the infected Δ*dcl2* strain for extended periods of sub-culturing. PCR analysis of the extracted viral RNA confirmed the loss of the recombinant viral EGFP gene sequence in the Δ*rdr* mutant strains as early as the first sub-culture and persistence of the EGFP gene sequence in the Δ*dcl2* strain. Similary, DI RNAs were readily detected in the virus-infected parental strains and in the individual and triple *rdr* gene disruption strains, but not in the virus-infected Δ*dcl2* strain (data not shown). Thus, these extensive sets of infection sub-culturing experiments (performed in triplicate) failed to reveal any observable contribution of the *rdr* genes to RNA silencing-mediated promotion of viral RNA recombination.

### 3.7. Contribution of RdRPs to transposon silencing

We next investigated whether knocking out one or more of the *rdr* genes affected the expression of transposable elements. Because the *C. parasitica* genome contains >100 transposon and transposon-like consensus sequences of >500 bp (see Methods), RNA-seq was performed to investigate whether global transposon expression was altered in the *rdr* gene knockouts. To identify and retrieve transposon sequences, we used a series of queries to search the annotated *C. parasitica* genome on the JGI website (see Methods), which identified 104 known or putative transposable elements. Illumina sequencing reads representing the entire transcriptome in Δ*rdr1/2/*3, Δ*rdr1/2* and Δ*rdr4* and the parental DK80 strain were then used in RNA-seq mappings to these transposon sequences and expression analysis in CLCBio’s Genomics Workbench. About 0.1% of the total reads in each strain mapped to the transposon sequences (not shown), and RNA-seq detected expression for 60 of the transposon sequences in at least one of the strains. The RNA-seq results indicated that expression of these transposon sequences was very variable: some sequences yielded <10 mapped reads, whereas others resulted in >9,000 mapped reads. However, only ∼20 transposable elements displayed significant expression (i.e., >100 mapped reads per sequence), none of which showed significant (i.e., ≥2 fold) up- or down-regulation in any of the *rdr* gene knockout strains (results for Δ*rdr1/2/3* and Δ*rdr4* are shown in Table S1). Thus, disruption of the *rdr* genes had no apparent effect on transposon transcript accumulation.

## Discussion

Molecular genetic characterization of the RNA silencing components encoded by *C. parasitica* has clearly demonstrated that the role of RNA silencing as an antiviral defense response observed in plants and invertebrates extends to the fungi. However, only a subset of the RNA silencing apparatus, a single Dicer protein, DCL2, and a single Argonaute protein, AGL2, are required for this important function. The antiviral defense response functions independently of the additional Dicer gene, *dcl1,* and three additional Argonaute genes, *agl1*, *agl3* and *agl4*, encoded by this fungus. The extensive analysis of the *C. parasitica* RdRP genes described here failed to find any evidence for contributions by any of these components to the antiviral defense response.

The four *C. parasitica rdr* genes fit into the three clades of RdRPs found in most filamentous fungi [51] that correspond to the three *N. crassa* RdRP genes. The *C. parasitica rdr4* gene clustered in the clade containing *N. crassa* RRP-3, for which no function has been identified. Interestingly, *rdr4* displayed alternative splicing, resulting in each case in transcripts whose products lacked an intact conserved RdRP domain, including the essential DXDGD motif. Thus, *rdr4* appears to be a non-functional RdRP pseudogene. In this regard, there is considerable evidence for the loss and expansion of RNA silencing genes in fungi [52], e.g., the apparent degeneration of the *Aspergillus nidulans* QDE-1 ortholog [51] and truncation of *A. nidulans* Dicer DCL-1 and Argonaute SMS-2 orthologs [17].

Of the three *C. parasitica* RdRP genes with intact RdRP motifs, *rdr3* clustered with *N. crassa* SAD-1, required for meiotic silencing of unpaired DNA (MSUD), while both *rdr1* and *rdr2* clustered with *N. crassa qde-1*, involved in vegetative silencing or Quelling. Since the single Dicer gene *dcl2* and single Argonaute gene *agl2* required for the antiviral defense response in *C. parasitica* are orthologs of the *N. crassa* Dicer (*dcl-2*) and Argonaute (*qde-2*) genes involved in Quelling, the *rdr1* and *rdr2* genes would logically be the most likely candidates to be involved in possibly amplifying the antiviral response.

Examination of RdRP gene expression levels in response to hypovirus infection showed that *rdr1* and *rdr2* transcript levels did not increase following virus infection as was observed for the *dcl2* and *agl2* antiviral defense genes. Surprisingly, the accumulation of *rdr3* and *rdr4* transcripts did significantly increase in response to virus infection (Fig. 4). Moreover, as reported for *dcl2* and *agl2* [20], [21], *rdr3* and *rdr4* expression was further increased following infection of the Δp29 virus lacking the suppressor of RNA silencing p29 (Fig. 4). Altered patterns of *rdr3* and *rdr4* expression were also observed in the Δ*dcl2* and Δ*agl2* mutant strains (Fig. 5). Interestingly, expression of the *N. crassa rdr4* ortholog *rrp3* increased 30-fold in response to dsRNA, leading Choudhary et al. [53] to propose that *rrp3* may play a role in the formation of viral or transposon dsRNA.

While the transcript accumulation results indicated some level of interaction at the expression level between the antiviral defense genes and at least *rdr3* and *rdr4*, supporting evidence for a role of the *rdr* genes in the antiviral defense response was not forthcoming from extensive disruption analysis of the *rdr* genes. No alterations in either virus-induced symptoms or in virus accumulation levels, as observed for *dcl2* and *agl2* disruption mutants, were detected in single or multiple *rdr* gene disruption mutant strain including the triple *rdr1*, *rdr2*, *rdr3* mutant. Thus, we conclude that *C. parasitica* is able to mount a robust RNA-silencing antiviral defense response without contributions from the encoded *rdr* genes. This clearly contrasts with the observations that RdRPs serve to amplify the RNA silencing antiviral defense response in plants [14], [22], [23] and in *C. elegans* [24]–[26]. However, the very large induction in *C. parasitica dcl2* expression in response to CHV1-EP713 infection [20], [21] may preclude the need for RdRP amplification of the antiviral defense signal.

We recently reported that the *C. parasitica* Dicer and Argonaute proteins involved in antiviral defense also contribute to viral RNA recombination to produce viral defective interfering (DI) RNAs and to delete non-viral RNA sequences from viral-based expression vectors [20], [21]. DI RNA formation and vector RNA stability was indistinguishable in parental, individual *rdr* mutant and triple *rdr* mutant strains. Thus, the *rdrs* also appear not to contribute to this RNA-silencing antiviral defense-related effect.

The accumulation of transposable element transcripts and fragments have been reported to be modulated by RNA-silencing pathways in several filamentous fungi [12], [13], [15], [32], [54], [55], and specific roles for RdRPs in this process have been reported for *Cryptococcus neoformans* [12], [32], *Mucor circinelloides* [13] and *N. crassa* [54]. Illumina sequencing of *C. parasitica* small RNAs isolated from wild type and Δ*dcl2*/Δ*agl2* strains identified the presence of DCL2/AGL2-derived transposable element-specific siRNAs (data not shown), indicating targeting of TE RNA by the antiviral defense RNA-silencing pathway. However, no significant increase in TE transcript accumulation was observed in RNA-seq analysis of the *rdr* knockout strains, including the triple knockout strain (Table S1). In this regard, Nolan et al. [11] reported that silencing of the introduced LINE1-like transposon *Tad* in *N. crassa* required one of the two Dicer genes and Argonaute gene *qde-2*, but surprisingly, not the RdRP gene *qde1*. The mechanism leading to the generation of TE-specific siRNAs in *C. parasitica* requires additional investigation.

The determination that *C. parasitica rdr* genes do not play a significant role in the RNA-silencing antiviral defense response, the primary aim of the study, is important information with bearing on future investigations. Disruption of individual and combinations of *rdr* genes also resulted in no observable changes in growth, colony morphology, asexual sporulation or virulence on chestnut. Similar results were observed for individual argonaute disruption mutants and individual and double dicer gene-disruption mutants in the absence of virus infection. Thus, the RNA-silencing components in *C. parasitica* also appear not to contribute to developmental, metabolic or morphological functions during vegetative growth, as has been reported for *dcl2* and *rdr3* disruption mutants of *Trichoderma atroviride* [56]. Reports of a role for RNA-silencing pathways in stress responses have been mixed. The *N. crassa gdq-1 rdr* and dicer gene mutant strains were reported to be more sensitive to histidine and DNA-damaging agents, such as hydroxyurea [31], while *C. neoformans* RNA-silencing mutants showed no alterations in stress responses [12]. The *C. parasitica* individual and combined *rdr* mutants and Δ*dcl2* failed to show any altered response to histidine or hydroxyurea (Table 2) and appear not to be involved in stress responses. However, the extensive characterization reported here does not eliminate possible roles for the *C. parasitica rdrs* in co-suppression or meiotic silencing, a determination that will require the development of additional experimental capabilities with this fungus.

## Supporting Information

Figure S1 **Disruption of** ***C. parasitica rdr1*** **in EP155.** The 4957 bp fragment (850 bp left arm + *rdr1* gene 3763 bp + 344 bp right arm) is replaced by pro-trpC-*hph* cassette (2172 bp). The primer pair R1f (310 bp upstream of the disruption fragment) and R1r (727 bp downstream of the disruption fragment) was designed to confirm the disruption yielding a 6.0 kb fragment from the wild-type strain and a 3.2 kb fragment from Δ*rdr1*.
(TIF)

Figure S2 **Disruption of** ***C. parasitica rdr2*** **.** Δ*rdr2* was generated by the replacement of the 1.5 kb fragment in the *rdr2* gene with a 3-kb *hph* cassette. The disruption confirmation primer R2f/R2r pair amplified the intact *rdr2* gene (∼4.1 kb) in the wild-type strain and a 5.7 kb fragment in Δ*rdr2*.
(TIF)

Figure S3 **Disruption of** ***C. parasitica rdr4*** **gene.** A fragment including 37 bp upstream of *rdr4* gene start codon and 3836 bp of the *rdr4* gene containing two alternative spliced introns (showing in dashed lines in the wild type strain) was replaced with the l*oxP*-flanked *hph* cassette (1.4 kb). Primer pair R4f/R4r yielded a 2.4 kb PCR product for Δ*rdr4* versus a 4.8 kb fragment for the wild-type strain.
(TIF)

Table S1 **RNA-seq results for identified transposon element sequences for strains** ***Δrdr1/2/3*** **and Δ** ***rdr4*** **.**
(XLSX)
