# Supplementary figures and images for: Characterizing the Roles of Cryphonectria parasitica RNA-Dependent RNA Polymerase-Like Genes in Antiviral Defense, Viral Recombination and Transposon Transcript Accumulation
Source: PLoS One. 2014 Sep 30;9(9):e108653. doi: 10.1371/journal.pone.0108653 (PMC4182546; doi:10.1371/journal.pone.0108653)

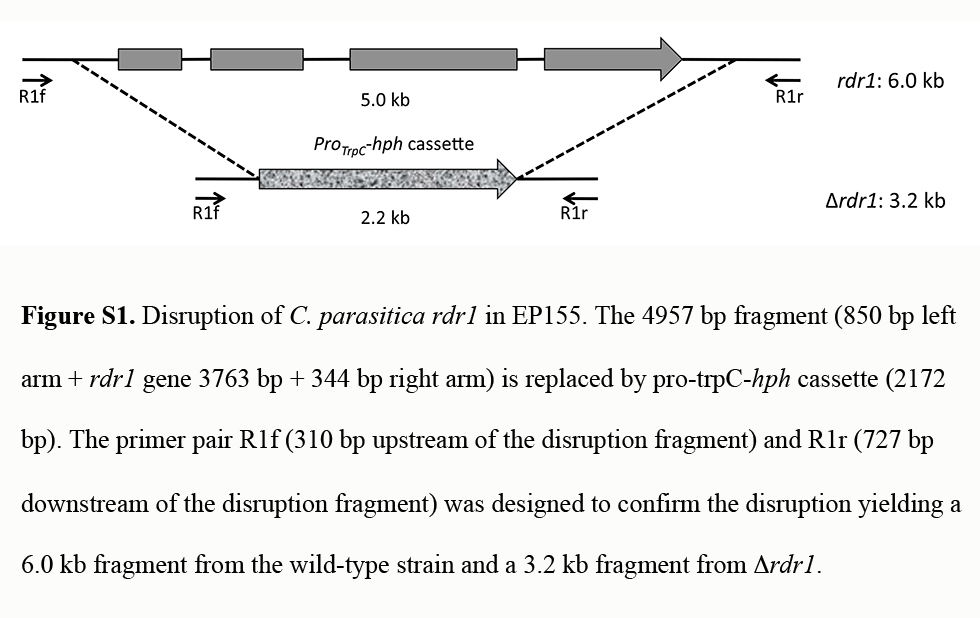

Supplement: Figure S1 — Disruption of C. parasitica rdr1 in EP155. The 4957 bp fragment (850 bp left arm + rdr1 gene 3763 bp + 344 bp right arm) is replaced by pro-trpC-hph cassette (2172 bp). The primer pair R1f (310 bp upstream of the disruption fragment) and R1r (727 bp downstream of the disruption fragment) was designed to confirm the disruption yielding a 6.0 kb fragment from the wild-type strain and a 3.2 kb fragment from Δrdr1. (TIF) [file pone.0108653.s001.tif]

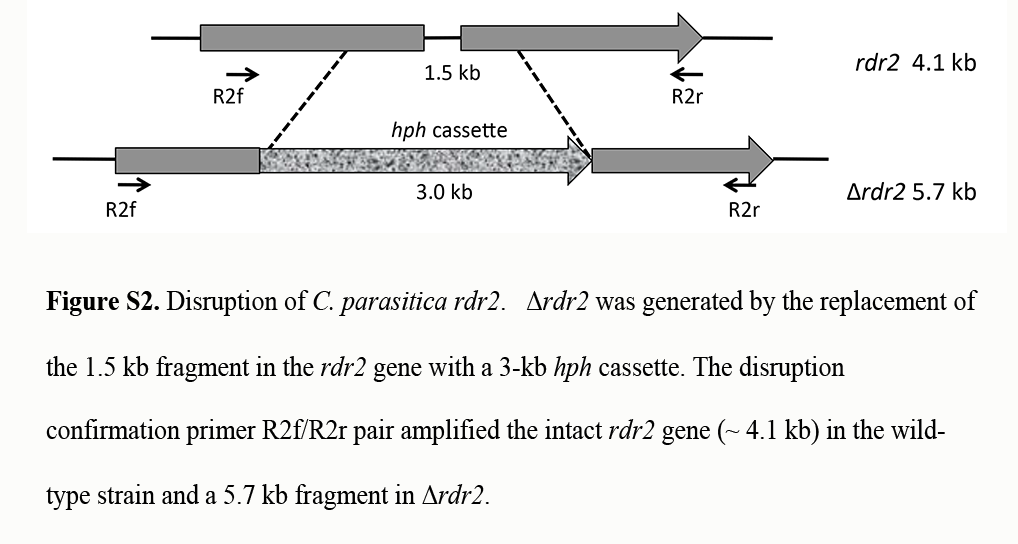

Supplement: Figure S2 — Disruption of C. parasitica rdr2 . Δrdr2 was generated by the replacement of the 1.5 kb fragment in the rdr2 gene with a 3-kb hph cassette. The disruption confirmation primer R2f/R2r pair amplified the intact rdr2 gene (∼4.1 kb) in the wild-type strain and a 5.7 kb fragment in Δrdr2. (TIF) [file pone.0108653.s002.tif]

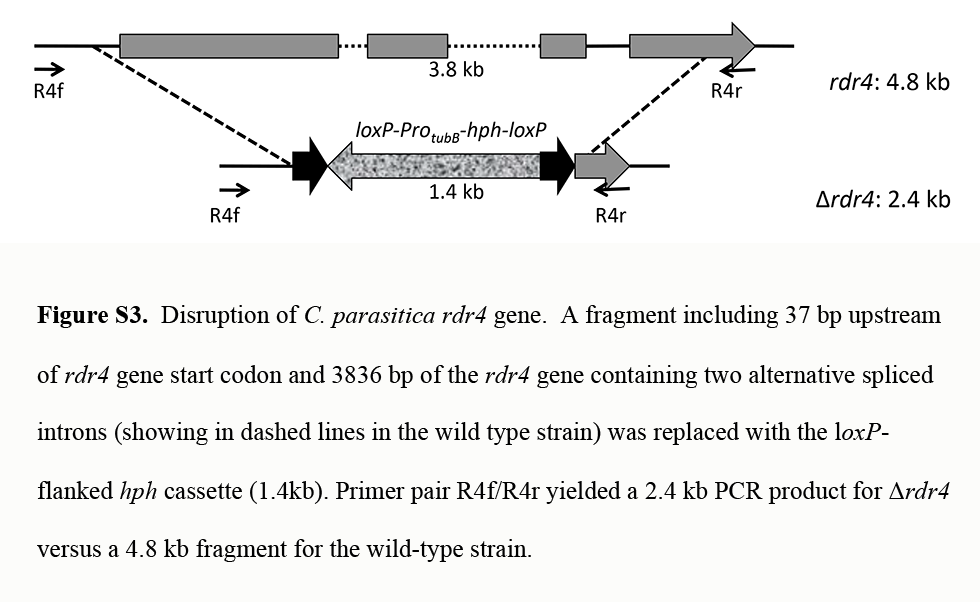

Supplement: Figure S3 — Disruption of C. parasitica rdr4 gene. A fragment including 37 bp upstream of rdr4 gene start codon and 3836 bp of the rdr4 gene containing two alternative spliced introns (showing in dashed lines in the wild type strain) was replaced with the loxP-flanked hph cassette (1.4 kb). Primer pair R4f/R4r yielded a 2.4 kb PCR product for Δrdr4 versus a 4.8 kb fragment for the wild-type strain. (TIF) [file pone.0108653.s003.tif]
